# Characterization of the Primary Human Trophoblast Cell Secretome Using Stable Isotope Labeling With Amino Acids in Cell Culture

**DOI:** 10.3389/fcell.2021.704781

**Published:** 2021-09-14

**Authors:** Fredrick J. Rosario, Sammy Pardo, Trond M. Michelsen, Kathryn Erickson, Lorna Moore, Theresa L. Powell, Susan T. Weintraub, Thomas Jansson

**Affiliations:** ^1^Division of Reproductive Sciences, Department of OB/GYN, University of Colorado Anschutz Medical Campus, Aurora, CO, United States; ^2^Department of Biochemistry and Structural Biology, University of Texas Health Science Center at San Antonio, San Antonio, TX, United States; ^3^Division of Obstetrics and Gynecology, Department of Obstetrics Rikshospitalet, Oslo University Hospital, Oslo, Norway; ^4^Section of Neonatology, Department of Pediatrics, University of Colorado Anschutz Medical Campus, Aurora, CO, United States

**Keywords:** placenta, pregnancy, maternal-fetal exchange, mass spectrometry relative protein synthesis rates, proteomics

## Abstract

The placental villus syncytiotrophoblast, the nutrient-transporting and hormone-producing epithelium of the human placenta, is a critical regulator of fetal development and maternal physiology. However, the identities of the proteins synthesized and secreted by primary human trophoblast (PHT) cells remain unknown. Stable Isotope Labeling with Amino Acids in Cell Culture followed by mass spectrometry analysis of the conditioned media was used to identify secreted proteins and obtain information about their relative rates of synthesis in syncytialized multinucleated PHT cells isolated from normal term placental villus tissue (*n* = 4/independent placenta). A total of 1,344 proteins were identified, most of which have not previously been reported to be secreted by the human placenta or trophoblast. The majority of secreted proteins are involved in energy and carbon metabolism, glycolysis, biosynthesis of amino acids, purine metabolism, and fatty acid degradation. Histone family proteins and mitochondrial proteins were among proteins with the slowest synthesis rate whereas proteins associated with signaling and the plasma membrane were synthesized rapidly. There was a significant overlap between the PHT secretome and proteins known be secreted to the fetal circulation by the human placenta *in vivo*. The generated data will guide future experiments to determine the function of individual secreted proteins and will help us better understand how the placenta controls maternal and fetal physiology.

## Introduction

The human placenta constitutes the interface between the maternal and fetal circulations and performs a wide array of functions, including nutrient and oxygen transport and secretion of hormones and exosomes (Costa, 2016). Hormones secreted by the placenta into the maternal circulation are believed to mediate maternal physiological adaptions to pregnancy. For example, animal studies have shown that placental lactogen promotes maternal β-cell proliferation and increases glucose-stimulated insulin secretion (Brelje et al., 1994; Sorenson and Brelje, 1997; Kim et al., 2010; Baeyens et al., 2016), and placental growth hormone (pGH) induces skeletal muscle insulin resistance in the mother (Barbour et al., 2004). Moreover, normal fetal growth and development is critically dependent on a well-functioning placenta, and most common pregnancy complications, including intrauterine growth restriction (IUGR), stillbirth, and preeclampsia, are caused by abnormal development and/or function of the placenta (Sibley et al., 2005; Mifsud and Sebire, 2014; Fisher, 2015; Sircar et al., 2015). Thus, a better understanding of the mechanisms by which the human placenta regulates fetal development and maternal physiology will provide insights into the pathophysiology of pregnancy complications and how changes in placental function determines life-long health.

To allow early prediction of development of pregnancy complications caused by altered placental function and to design new intervention strategies targeting the placenta, sensitive biomarkers for placental function that can be measured using a minimally invasive approach, preferably in a maternal blood sample, are required. Unfortunately, no such approach is currently available, and the search for biomarkers for early detection of serious pregnancy complications has been disappointing. Indeed, recent systematic reviews and meta-analyses suggest that none of the currently available biomarkers predict IUGR or preeclampsia with sufficient sensitivity to be used in routine clinical practice (Conde-Agudelo et al., 2013; Chaiworapongsa et al., 2014; Wu et al., 2015; Zhong et al., 2015). One of the problems using maternal plasma proteomics to identify biomarkers for placental function is that the contribution of the placenta to the maternal plasma proteome is largely unknown. Characterization of the proteins synthesized and secreted by the placenta or primary human trophoblast (PHT) cells would help address this gap in knowledge and allow a more focused approach in the search for biomarkers for placental function.

To date, analyses of protein levels in the placenta have been performed primarily using two-dimensional (2D) PAGE (He et al., 2014) or surface-enhanced laser desorption/ionization (SELDI) mass spectrometry (Luciano-Montalvo et al., 2008). The SELDI results indicated alterations in protein expression patterns but were not able to provide comprehensive identification of individual proteins (Batorfi et al., 2003). Advances in technology have made it possible to apply mass spectrometry to a wide range of cell culture-based studies (Aebersold and Mann, 2003). Stable Isotope Labeling with Amino Acids in Cell Culture, or SILAC, has emerged as a valuable proteomic technique (Ong et al., 2002, 2003). Using SILAC, cells representing two or more biological conditions can be cultured in growth media supplemented with specific unlabeled (“light”) or stable isotope-labeled (“heavy”) amino acids (usually lysine and arginine). The proteins being synthesized in these cell populations incorporate the corresponding “light” or “heavy” amino acids. In cells that are dividing, essentially 100% amino acid incorporation can be readily achieved, permitting relative protein quantification for two experimental conditions using light/heavy-labeled cultures. For cells that do not divide, such as syncytiotrophoblasts, or cells that divide very slowly, incorporation of label depends on the rate of protein synthesis, and, thus, inclusion of stable isotope labeled amino acids provides a powerful approach to obtain a relative measure of the rate of protein synthesis while at the same time identifying the proteins in the cells. Recent studies in mice (Abdulghani et al., 2019; Napso et al., 2021) and humans (Michelsen et al., 2019) suggest that analysis of the placental secretome/proteome can provide information on candidate biomarkers for pregnancy complications, and placental secretome regulates maternal islet cell mass and functions (Drynda et al., 2018). In addition, the results from characterization of human placental macrophage secretome suggest that proteins secreted by placental macrophages at term pregnancy are essential for protecting fetuses against various viral infections (Garcia et al., 2009). However, no discovery approach has been used to identify the proteins secreted by cultured PHT cells isolated from term placenta. To assess trophoblast protein synthesis rate, inform efforts to find novel protein biomarkers for trophoblast function, and identify trophoblast proteins that regulate maternal physiological adaptations to pregnancy and influence fetal development and growth, we employed a SILAC-based mass spectrometric approach to characterize the secretome of PHT cells.

## Materials and Methods

### Placental Collection

Healthy women with normal term pregnancies (>37 weeks of gestation) delivered by Cesarean section were recruited following written informed consent. The exclusion criteria were: smoking; use of illicit drugs; concurrent diseases, such as diabetes and hypertension; and development of pregnancy complications including gestational diabetes, pregnancy-induced hypertension and preeclampsia. The Institutional Review Board at the University of Texas Health Science Center at San Antonio approved the protocol (HSC20100262H); study personnel provided the de-identified samples and clinical information used in this study.

### Stable Isotope Labeling With Amino Acids in Cell Culture

Primary human trophoblast cells were isolated using a well-established method involving sequential trypsin digestion and Percoll centrifugation (Kliman et al., 1986) as described in Supplementary Material. For SILAC labeling, PHT cells were cultured in DMEM/F12 media containing [^2^H_4_] L-lysine (700 μM) and [^13^C_6_] L-arginine (700 μM) (K4R6, Cambridge Isotope Laboratories, Inc., Andover, MA, United States) starting at 18 h following plating. In parallel, PHT cells were grown in the same media containing unlabeled L-lysine and L-arginine instead of isotopically labeled variants; media was refreshed every 24 h. At 90 h, cells were washed five times with phosphate-buffered saline to remove excess bovine serum proteins (Pellitteri-Hahn et al., 2006; Stastna and Van Eyk, 2012) and then incubated in serum-free labeled and unlabeled media for a period of 24 h. At 114 h and following 24-h culture in serum-free media, the conditioned media was collected, and human chorionic gonadotropin (hCG) was measured (Supplementary Figure 1). In addition, conditioned media was processed and analyzed by mass spectrometry as described in detail in Supplementary Material.

### Bioinformatics

Gene Ontology term enrichment analysis was performed using the DAVID bioinformatics resource (Huang da et al., 2009). The biological processes and molecular functions of secreted proteins were categorized by Ingenuity Pathway Analysis (IPA; Kramer et al., 2014). Prediction of subcellular localization and exosome comparison were obtained using Functional Enrichment Analysis Tool^1^ and the Exo Carta database,^2^ respectively.

### Statistical Analysis

Analyses were performed using GraphPad Prism 6 software. Results were statistically significant if *p* < 0.05.

## Results

### Proteins Identified in the PHT Cell Secretome

Proteins in the PHT cell conditioned media were separated by 1D SDS PAGE, and the gel lanes were excised into six slices and subjected to in-gel digestion followed by HPLC-electrospray ionization tandem mass spectrometry analysis. Four independent biological replicates (conditioned media of PHT cells isolated from four separate placentas) were analyzed. There were a total of 1,344 secreted proteins identified by at least one peptide spectrum match among the four samples (Supplementary Table 1). It is interesting to note that among the PHT secreted proteins were many related to nutrient transport, including phospholipid transfer protein (PTLP), vitamin D binding protein (GC), protein transport protein [SEC23A or B (Sec23 homolog A or B); COPII (Coat Complex Component); and phosphatidylinositol transfer protein (PITPN)] (Supplementary Table 1). In addition, numerous members of the serpin family were found in the PHT secretome (Supplementary Table 2).

### Functional Characterization

**FIGURE 1:**
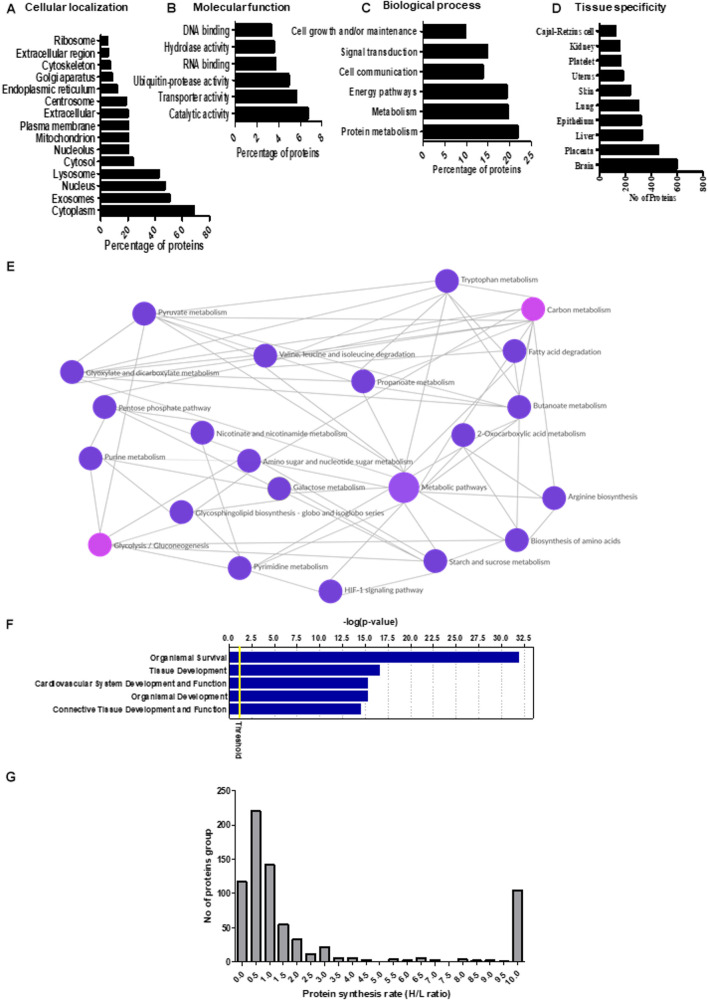
**(A–D)** Gene ontology and Kyoto Encyclopedia of Genes and Genomes (KEGG) pathway analysis of the primary human trophoblast (PHT) secretome. The proteins identified in the PHT secretome were analyzed by Functional Enrichment Analysis Tool (www.funrich.org), which provides prediction of subcellular localization prediction software to predict the **(A)** cellular localization, **(B)** molecular function, and **(C)** biological processes of proteins in the secretome. **(D)** The identified proteins in the PHT secretome were analyzed by Gene Ontology term “tissue specificity” annotation using DAVID Bioinformatics Resources 6.8. **(E)** KEGG pathway analysis of the PHT secretome. The KEGG database was used to identify enriched pathways against the background of *Homo sapiens* for the PHT secreted proteins. KEGG pathway analysis shows enrichment of proteins related to carbon metabolism (including glycolysis, biosynthesis of amino acids), purine metabolism and fatty acid degradation in the PHT secretome. (Reprinted with permission from Kyoto Encyclopedia of Genes and Genomes, http://www.kegg.jp/kegg/kegg1.html). **(F)** Ingenuity Pathway Analysis (IPA) assignment of physiological process imputed from the PHT cell secretome. The list of PHT secreted proteins was submitted to IPA to find statistically enriched physiological functions. A function is significantly enriched when the percentage of proteins annotated with this function is above the proportion of annotated protein in the secretome (threshold *p*-value <0.05). The graph shows the top five enriched physiological functions reported by IPA according to the mapped secreted protein lists. Ordinates: -log (*p*-value) correspond to the *p*-values obtained by a Fisher test with Benjamini–Hochberg correction. **(G)** The distribution of relative protein synthesis rates in the PHT secretome. The ratio of “heavy” to “light” protein forms ranged from 10 to 0.01, with a median of 0.84. The protein synthesis rate distribution in the PHT secretome is skewed toward smaller values, reflecting the larger proportion of proteins with slower synthesis rates.

Prediction of the subcellular localization of the secreted proteins indicated that the top-ranked cellular compartments for the PHT secretome were cytoplasm, extracellular vesicles, nucleus, and lysosomes (Figure 1A). Of the 1,344 identified proteins in the PHT cell secretome, 50% were predicted to be associated with extracellular vesicles (Figure 1A). These findings are consistent with recent reports that trophoblast-derived extracellular vesicles play a key role in placental orchestration of pregnancy and maternal immune sensing of the fetus (Stefanski et al., 2019). Furthermore, 68% of secreted proteins were associated with the cytoplasm. It cannot be excluded that some of these proteins represent unspecific leakage of PHT cell cytoplasmic proteins into the conditioned media. However, these proteins may be secreted via unconventional protein-secretory pathways, possibly mediated by Golgi or endosomal export mechanisms (Nickel and Rabouille, 2009). Alternatively, cytoplasmic proteins may be secreted after being incorporated into the membrane or the intravehicular space of extracellular vesicles released by PHT cells.

### Gene Ontology Analysis

The results of gene ontology annotation for the molecular function of the PHT secretome revealed that catalytic activity and transporter activity were the two predominant functional groups (Figure 1B). Moreover, processes such as metabolism, energy pathways and cell communications were among the top-ranked biological functions (Figure 1C).

Using the UP TISSUE tool in DAVID, we compared the protein distribution of the PHT secretome with tissue expression databases (Figure 1D) and found that large subgroups of proteins in the PHT secretome are associated with the brain (50%), placenta (38%), liver (28%), and epithelium (27%). Furthermore, using the Kyoto Encyclopedia for Genes and Genomes (KEGG), we found enrichment for pathways related to carbon metabolism that can be linked to trophoblast function, including glycolysis, biosynthesis of amino acids, purine metabolism, and fatty acid degradation (Figure 1E). For example, triosephosphate isomerase (TPI1) was secreted by PHT cells. TPI1 is known to catalyze the reversible interconversion of dihydroxyacetone phosphate and D-glyceraldehyde 3-phosphate; it plays an important role in glycolysis and is essential for efficient ATP production. Other proteins in the PHT secretome related to the citric acid cycle and ATP production are dihydrolipoamide dehydrogenase precursor (DLD), isocitrate dehydrogenase 1 (IDH1) and aconitase 1 (ACO1).

### Pathway Analysis

Pathway analysis of the PHT secretome revealed over-representation of various signaling mechanisms, including EIF2, eIF4, mTOR, and p70S6 kinases signaling and protein ubiquitination pathway (Table 1). IPA demonstrated that the PHT secretome was enriched for various physiological systems and functions (Figure 1F), including organismal survival (418 proteins), tissue development (306 proteins), cardiovascular system development and functions (263 proteins), organismal development (368 proteins) and connective tissue development and function (211 proteins). In the category “morphology of cardiovascular system,” catenin alpha-3 (CTTNA-3), heat shock protein family B (HSPFB-8) and prolyl isomerase 1A (FKBP 1A) are associated with cardiovascular development, all of which were enriched in the PHT secretome.

**TABLE 1:** Canonical pathway analysis of primary human trophoblast (PHT) secretome by Ingenuity Pathway Analysis (IPA).

**Top canonical pathways**	***p*-value^1^**	**Overlap**
EIF2 signaling	1.8 E-36	33%
Protein ubiquitination	3.8 E-35	29%
Regulation of eIF4 and p70S6K signaling	3.8 E-28	35%
mTOR signaling	1.7 E-20	26%
Clathrin mediated endocytosis signaling	1.0 E-17	25%

*^1^The *p*-values were calculated from hypergeometric tests based on the number of the overlapping molecular relations between the generated network and the canonical pathways stored in IPA.*

### Protein Synthesis Rate in the PHT Secretome

In addition to protein identification, the SILAC results provided information about the synthesis rate for 731 protein in the PHT secretome (Supplementary Table 3). The ratio of “heavy” to “light” protein forms ranged from 0.01 to 10, with a median of 0.84 (Figure 1G). We arbitrarily defined proteins with an H/L ratio of ≥1 as fast synthesis proteins. The distribution of ratios is skewed toward lower ratios, reflecting a greater abundance of proteins with slower synthesis rates. This is likely related to the general slow synthesis of proteins that are components of non-dividing cells (Savas et al., 2012), including PHT cells. Twenty-two histone family proteins were among the slowest to incorporate label (Supplementary Table 4). The relationship between protein synthesis rate and subcellular location of the PHT secretome was examined using Functional Enrichment Analysis Tool (Supplementary Figure 2). Proteins in the cytoplasm, extracellular vesicles, lysosome and nucleus were found to be enriched in both the slow- and fast-synthesis classes (Supplementary Figure 2). A trend was apparent that proteins with a relatively slower synthesis rate were associated with mitochondria, nucleosomes, nucleoplasm, proteasome complex and ribosomes.

We found that secreted proteins from PHT cells associated with distinct biological processes exhibited similar trends with respect to protein synthesis rates (Supplementary Figure 3). For example, proteins involved in protein metabolism, signal transduction, cell communication, cell growth and maintenance were overrepresented in the group of proteins with fast rates of synthesis. In contrast, proteins involved in energy pathways, metabolism and regulation of gene expression and epigenetics were among those with slower synthesis. We also examined the relative synthesis rates of different subunits within the same protein complex. For most complexes, such as the proteasome and ATP synthase, subunits had similar synthesis rates. This suggests that the synthesis and degradation of the different subunits in the PHT secretome are coordinated. However, ribosomal subunits were synthesized at different rates. Ribosomal proteins are synthesized in the cytoplasm and subsequently assembled into the large and small ribosomal subunits in the nucleus and nucleolus where they interact with a variety of assembly proteins and ribosomal RNA before they are released back into the cytoplasm where they mediate protein synthesis. As shown in Supplementary Table 3, synthesis of 12 of 17 identified ribosomal proteins was relatively slow.

### Associations Between the PHT Cell Secretome and the Placental Proteome Secreted Into the Fetal Circulation

**FIGURE 2:**
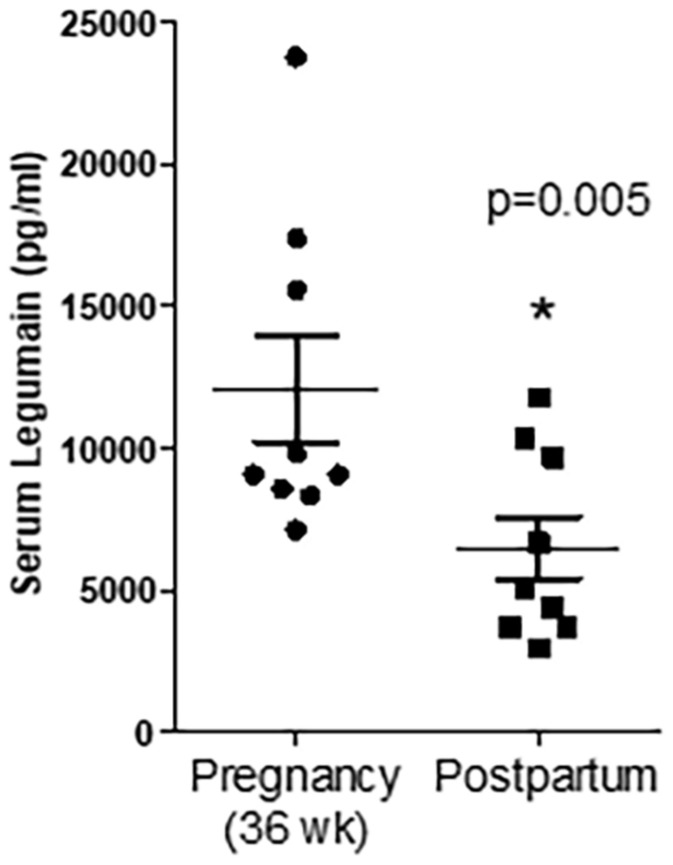
Serum concentration of legumain at 36 weeks of pregnancy and the third week of postpartum. Values are mean + SEM. *P < 0.05 considered significant; Student’s *t*-test, *n* = 9/each group.

Factors secreted from the human placenta are believed to be critical for fetal development (Bonnin et al., 2011; Behura et al., 2019a). Using an aptamer-based proteomic approach, we previously reported that 341 proteins are specifically secreted by the human term placenta into the fetal circulation, as evidenced by significantly higher concentrations in the umbilical vein compared to the umbilical artery (Michelsen et al., 2018). In the current study, we found that 47 of the 341 proteins secreted into the fetal circulation by the human placenta *in vivo* were also secreted by cultured PHT cells (Table 2). Moreover, as a proof of concept we quantified the abundance of legumain (one of the placental factors secreted by PHT cells) in paired serum samples collected from women during her pregnancy (36 weeks of pregnancy) and postpartum (3rd week of postpartum). We found that serum legumin level was higher in 36 weeks of pregnancy as compared to postpartum (Figure 2).

**TABLE 2:** Proteins secreted by PHT cells *in vitro* and by the human placenta into the fetal circulation *in vivo*.

**Uni Prot ID**	**Target full name**
O43278	Kunitz-type protease inhibitor 1
O43464	Serine protease HTRA2, mitochondrial
P00533	Epidermal growth factor receptor
P01023	Alpha-2-macroglobulin
P01024	Complement C3
P01034	Cystatin-C
P01215	Glycoprotein hormones alpha chain
P02649	Apolipoprotein E
P02751	Fibronectin
P04179	Superoxide dismutase [Mn], mitochondrial
P05155	Plasma protease C1 inhibitor
P05362	Intercellular adhesion molecule 1
P06396	Gelsolin
P07339	Cathepsin D
P0DMV8	Heat shock 70 kDa protein 1A
P12277	Creatine kinase B-type
P12830	Cadherin-1
P13987	CD59 glycoprotein
P15586	N-acetylglucosamine-6-sulfatase
P28799	Granulins
P30040	Endoplasmic reticulum resident protein 29
P33151	Cadherin-5
P36222	Chitinase-3-like protein 1
P36955	Pigment epithelium-derived factor
P42702	Leukemia inhibitory factor receptor
P61626	Lysozyme C
P61769	Beta-2-microglobulin
P68871	Hemoglobin subunit beta
P69905	Hemoglobin subunit alpha
Q02487	Desmocollin-2
Q03167	Transforming growth factor beta receptor type 3
Q07954	Prolow-density lipoprotein receptor-related protein 1
Q08380	Galectin-3-binding protein
Q12841	Follistatin-related protein 1
Q14126	Desmoglein-2
Q15582	Transforming growth factor-beta-induced protein ig-h3
Q15828	Cystatin-M
Q99988	Growth/differentiation factor 15
Q9NZ08	Endoplasmic reticulum aminopeptidase 1
Q9UBR2	Cathepsin Z

## Discussion

In the current study, we cultured PHT cells isolated from human term placenta using a widely-accepted standard protocol in which isolated cytotrophoblast cells form syncytial islands in culture (Kliman et al., 1986); and this is considered to be a physiologically relevant model to study the syncytiotrophoblast, the transporting epithelium of the human placenta. This is the first report of utilizing SILAC as a part of an effort to identify proteins secreted by syncytialized PHT cells, which do not divide in culture. The majority of proteins in the PHT secretome have not previously been reported to be secreted by the human placenta or trophoblast. Our results demonstrate the feasibility of using SILAC to characterize the PHT cell secretome and we present novel information. Conventional SILAC requires extensive metabolic labeling of proteins, and, therefore, is difficult to apply to cells that do not divide in culture (Spellman et al., 2008).

The SILAC approach labels any protein that is newly synthesized during the time period when label is present without preference for certain subgroups of proteins. In cells that are dividing, essentially 100% amino acid incorporation/coverage can be readily achieved, for example it has been reported that complete label incorporation occurred after five doublings in a range of cell lines (Ong et al., 2002). On the other hand, in non-dividing cells, such as primary neurons, incorporation is slow (Zhang et al., 2014). Because PHT cells do not divide, in the current study we incubated PHT cells in label from 18 to 114 h in culture to maximize label incorporation. We collected conditioned media for characterization of the PHT cell secretome the last 24 h in culture when trophoblast cells have formed a syncytium. Thus, the secretome we report represents proteins secreted by the syncytiotrophoblast. Because the syncytiotrophoblast constitutes the transporting and hormone producing epithelium of the human placenta and is the predominant cell type in the human placenta at term, we believe that it is likely that secreted proteins from the human placenta *in vivo* predominantly originate from this cell type. This provided the rationale for focusing on the secretome of the syncytiotrophoblast in the current study.

The detection in the PHT secretome of chorionic gonadotrophin subunit alpha (CGA), one of the subunits of hCG, which is a well-known marker for trophoblast differentiation and function (Posillico et al., 1985; Fournier et al., 2015), validates our approach and confirms the trophoblast origin of the secreted proteins. hCG acts on the uterine environment via the luteinizing hormone/hCG receptor and exerts autocrine effects, promoting differentiation, and migration of trophoblasts, and paracrine effects on the maternal endometrium (Shi et al., 1993). Fibronectin (FN) was another of the 1,344 proteins in the PHT secretome, in agreement with previous reports (Yuehong et al., 2001). FN is a member of a family of high molecular weight extracellular matrix glycoproteins that has been characterized as “trophoblast glue” and is highly abundant in regions rich in extravillous trophoblasts (Mercorio et al., 2006). Growing evidence suggests that placental and cord FN levels are elevated in preeclampsia (Uzun et al., 2010) and in recurrent pregnancy loss, and could be a potential candidate biomarker to assess placental function. PTLP secreted by trophoblasts is believed to be important for HDL assembly and regulation of maternal-fetal cholesterol transfer (Scholler et al., 2012). Also in the PHT secretome was Vitamin D binding protein, which is one of the key biomolecules involved in stimulation of calcium absorption for sufficient fetal bone mineral accrual and enhancing systemic and local maternal tolerance to paternal and fetal alloantigen (Karras et al., 2018). SEC23A and SEC23B are components of the coat protein complex II (COPII) which promotes the formation of transport vesicles from the endoplasmic reticulum; these proteins were found to be secreted by PHT cells. Both SEC23A and SEC23B are required for embryo morphogenesis, neural tube closure (Zhu et al., 2015), craniofacial chondrocyte maturation (Lang et al., 2006), and placental development.

Serpins are serine proteases that regulate an array of molecular pathways, such as inflammation, coagulation, fibrinolysis, complement activation, and phagocytosis; they can also be linked to trophoblast function. Serpin family members G1, B2, and E2 are necessary for proper circulatory function, and serpin deficiency is a risk factor for preeclampsia (Mondon et al., 2005; Severens-Rijvers et al., 2017). There are no reports in the literature of known function for SERPIN B9/E1/C1/B5/A1 and F1 in the placenta, even though they have been associated with inflammation, immune suppression, cell senescence, angiogenesis, coagulation, collagen biosynthesis, and invasion in other tissues (El Haddad et al., 2011; Heutinck et al., 2012; Jiang et al., 2017). Thus, secreted members of the serpin family of proteins are potentially novel regulators of trophoblast angiogenesis, invasion, cell senescence, and inflammation. Pathway analysis of the PHT secretome revealed over-representation of various signaling mechanisms, including EIF2, eIF4, mTOR, and p70S6 kinases signaling and protein ubiquitination pathway (Table 1). Possible interpretation of these findings is that mTOR signaling is involved in the regulation of trophoblast function or that the PHT secretome regulates mTOR signaling in other cells. There is a wealth of evidence demonstrating that the placental mTOR pathway responds to many growth-related signals, including amino acids, glucose, oxygen, folate, and growth factors, to regulate trophoblast mitochondrial respiration, nutrient transport, and protein synthesis, thereby influencing placental and fetal growth (Rosario et al., 2013, 2017, 2019).

Pathway analysis demonstrated that the PHT secretome was enriched for cardiovascular system development and function. These findings are consistent with the possibility that trophoblasts secrete proteins involved in maternal cardiovascular adaptation to pregnancy and/or fetal cardiovascular development. In support of this speculation, recent studies demonstrated that placental dysfunction may significantly contribute to the incidence of congenital heart diseases (Maslen, 2018). Moreover, defects in placentation are highly prevalent in embryonically lethal mouse mutants and placental defects correlate strongly with abnormal heart and vascular development (Perez-Garcia et al., 2018). However, a cause-and-effect relationship between placental secreted factors and cardiovascular development remains to be established.

Recent findings suggest that many proteins secreted from the trophoblast may remotely control the development of function of specific maternal and/or fetal tissues (Wu et al., 2017; Behura et al., 2019b). In support of this hypothesis, we recently found that 34 proteins were secreted by the placenta into the maternal circulation, as evidenced by significantly higher levels in uterine vein compared to radial artery (used to represent the uterine artery). The proteins secreted included placental growth factor, growth differentiation factor 15, and matrix metalloproteinase 12 (Michelsen et al., 2018). Similarly, 341 proteins were secreted by the placenta into the fetal circulation (Michelsen et al., 2018) based on significantly higher levels in the umbilical vein compared to the umbilical artery for samples collected simultaneously. It is also possible that the proteins identified in the PHT secretome reflect common functions between the placenta and other tissues. Both the liver and the placenta are tissues with high metabolic activity that share common pathways. For example, 1,4-alpha-glucan branching enzyme 1 (GBE1) identified in the PHT secretome is known to participate in glycogen biosynthesis by attaching a short glucosyl chain in an α-1,6-glucosidic link to a naked peripheral chain of nascent glycogen. GBE1 deficiency results in the accumulation of abnormal glycogen (polyglucosan) in placenta (Konstantinidou et al., 2008). The syncytiotrophoblast is the transporting and hormone-producing epithelium of the human placenta. Therefore, proteins that are associated with the tissue functional annotation term “epithelium” may have a role in placental transport, secretion, selective absorption, sensing, and protection similar to other epithelial cells.

The mechanisms by which proteins are secreted/released from the syncytiotrophoblast remain to be fully established but may involve any type of syncytiotrophoblast-derived extracellular vesicles, including small extracellular vesicles. Proteins in the PHT cell secretome may be secreted into the maternal and/or the fetal circulation. Whereas secretion of proteins synthesized by the syncytiotrophoblast into the maternal circulation is well established and includes hormones such as placental lactogen and pGH (Freemark, 2010), secretion of syncytial proteins into fetal blood is much less characterized. Although the fetal capillary endothelium is likely to restrict the passage of some proteins, there is ample evidence, including transfer of maternal IgG to the fetus and the transport of alpha fetoprotein into the maternal circulation, that large proteins do cross this barrier. Moreover, the observation that 50% of total exosomes in human fetal blood are of placental (syncytiotrophoblast) origin (Miranda et al., 2018) demonstrates that structures as large as ∼100 nm can cross the human placental capillary endothelium, although the mechanisms involved are largely unknown.

It is possible that the protein synthesis data can provide information on proteins that are subjected to short-term regulation because we speculate that a protein with a rapid synthesis rate is turned over rapidly and therefore more likely to be subjected to such regulation. It may be speculated that proteins involved in intracellular signaling and cell-to-cell communication should be short-lived for efficient and rapid fine-tuned regulation, while proteins that serve more structural functions in the cell are longer-lived in order to save energy that would be required for protein synthesis and degradation. We found that histone family members as well as proteins associated with mitochondria, ribosomes, gene expression, and epigenetics exhibited the slowest synthesis rates in the PHT secretome. This agrees with previous studies that showed slow synthesis rates of histone proteins in mouse embryonic neurons (Toyama et al., 2013) and is consistent with the fact that cultured PHT cells are fully differentiated, non-dividing, cells. Of these, proteins associated with mitochondria had the slowest synthesis rates, consistent with previous reports (Price et al., 2012; Dai et al., 2014). These findings are in general agreement with reports that distinct mechanisms of degradation may cause systematic differences in protein synthesis between cytosolic (Tai and Schuman, 2008) and membrane proteins (Avci and Lemberg, 2015). Our observation that proteins involved in regulation of gene expression and epigenetics have slow synthesis rates is consistent with previous reports in primary neurons (Mathieson et al., 2018). This trend is in line with a previous study in mice, where ribosomal proteins were found to turn over at slower rates compared to many other proteins in complexes (Price et al., 2010). Several ribosomal proteins exhibited a relatively fast synthesis rate in our study, including proteins associated with both the large (60S acidic ribosomal protein P2) and small (40SRps28, 40SRps3, and ubiquitin 40SRps27a) subunits. These ribosomal proteins often do not assemble into stable large ribosome units; an unassembled ribosome unit is rapidly degraded by the ubiquitin proteasome degradation pathways (Boisvert et al., 2012).

Our study has some limitations. For example, cell lysis may contribute to the characterized secretome, which is difficult to control for. Moreover, the expression “rate of protein synthesis” is a simplification because, rather than representing the actual rate of synthesis, it corresponds to the difference between actual synthesis of new protein during the study period minus any newly synthesized protein that has been degraded. The goal of this study was to characterize the syncytiotrophoblast secretome at term. Given the major changes in uteroplacental blood flow and in placental function and morphology across gestation, the findings reported in this study may not be representative for the syncytiotrophopblast secretome earlier in gestation. For example, it is well established that the maternal circulating levels of hCG, a protein synthesized and secreted predominantly by the syncytiotrophoblast, are very high at the end of the first trimester and subsequently decline to low levels at term, consistent with the notion that the first trimester and term syncytiotrophoblast secretomes are distinct.

## Conclusion

The placenta, and specifically the syncytiotrophoblast, is believed to release hormones into the maternal circulation that contribute to the maternal metabolic and cardiovascular adaptation to pregnancy. However, the identities of these hormones remain to be fully established. Emerging evidence in animal models demonstrates that specific placental factors secreted into the fetal circulation are critical for fetal development, however, it is not known if this occurs in humans. We demonstrate that SILAC-based mass spectrometry can be successfully applied to non-dividing PHT cells to characterize the human trophoblast secretome and obtain information about rates of protein synthesis. This dataset can be of value to identify novel placental proteins that regulate maternal physiology and/or fetal development. Identifying proteins in the PHT cell secretome does not provide information about whether these proteins are secreted into the maternal and/or fetal circulation. Additional studies of, for example, uteroplacental and umbilical concentrations gradients are needed to better understand what potential role these secreted proteins have *in vivo*. Although there is general consensus that factors in the maternal circulation that reflect placental function are ideal candidates for biomarkers for early diagnosis of pregnancy complications due to placental insufficiency, few, if any, maternal serum biomarkers are currently in clinical use. Our data defining the secretome of cultured PHT cells can serve as a starting point for a more targeted approach in the search for clinically useful maternal serum biomarkers for early detection of important pregnancy complications. The generated data will guide future experiments to gain insight into the function of individual secreted proteins and to better understand how the placenta controls maternal and fetal physiology. Moreover, because secreted proteins are often glycosylated, glycocapture-based proteomics for secretome analysis will allow a more focused approach to study classically secreted proteins from the trophoblast and the placenta, decreasing the complexity of the sample and circumventing the potential problem with proteins that have leaked out of the cells or released by ectodomain shedding.

## Data Availability Statement

The original contributions presented in the study are included in the article/Supplementary Material, further inquiries can be directed to the corresponding author/s.

## Ethics Statement

The studies involving human participants were reviewed and approved by the University of Texas Health Science Center at San Antonio. The patients/participants provided their written informed consent to participate in this study.

## Author Contributions

FR, SP, TP, SW, and TJ researched the data, designed the experiments, and wrote the manuscript. LM performed and collected the human serum samples. TM was responsible for four vessel sample data analysis and presentation of the data. FR, SP, and KE performed the experiments. All authors critically revised the manuscript for substantive content and approved the final version.

## Conflict of Interest

The authors declare that the research was conducted in the absence of any commercial or financial relationships that could be construed as a potential conflict of interest.

## Publisher’s Note

All claims expressed in this article are solely those of the authors and do not necessarily represent those of their affiliated organizations, or those of the publisher, the editors and the reviewers. Any product that may be evaluated in this article, or claim that may be made by its manufacturer, is not guaranteed or endorsed by the publisher.

## Footnotes

1
www.funrich.org

2
www.exocarta.org
