# Supplementary material for: Characterization of the Primary Human Trophoblast Cell Secretome Using Stable Isotope Labeling With Amino Acids in Cell Culture
Source: Front Cell Dev Biol. 2021 Sep 14;9:704781. doi: 10.3389/fcell.2021.704781 (PMC8476785; doi:10.3389/fcell.2021.704781)
Supplement: Supplementary file 2 [file Data_Sheet_1.docx]

**Supporting Information**

**Supplemental Table 1.** Proteins secreted from primary trophoblasts.

**Supplemental Table 2.** Members of the Serpin family of proteins identified in the PHT secretome.

**Supplemental Table 3.** Incorporation of stable isotope labeled K and R into proteins secreted from primary human trophoblasts.

**Supplemental Table 4.** Members of the histone family of proteins with low levels of stable isotope incorporation in the PHT secretome.

**Supplemental Table 5.**  Clinical characteristics of the study subjects.

**Supplemental Figure 1. Culturing PHT cells in SILAC media does not affect trophoblast cell viability, differentiation, or apoptosis.** (A) Cultured primary trophoblast cells were incubated in DMEM + F12 media with stable isotope labeled lysine and arginine (Lys4/Arg6) or DMEM + F12 media with unlabeled lysine and arginine. hCG secretion showed the expected increase over time in culture as a marker of syncytialization; the secretion profile did not differ between the two media. Values are mean + SEM for cultured PHT cells isolated from four different placentas. (B, C) Representative western blots of caspase-3 and syncytin expression in cell lysates of DMEM + F12 media with stable isotope labeled lysine and arginine (Lys4/Arg6) or DMEM + F12 media with unlabeled lysine and arginine cells. Equal loading was performed.

**Supplemental Figure 2**. **Gene Ontology (GO) analysis of fast and slow synthesis proteins of Primary Human Trophoblast secretome**. (A) The identified fast and slow synthesis proteins in the PHT secretome were analyzed by Functional Enrichment Analysis Tool ([www.funrich.org](http://www.funrich.org)), a subcellular localization predication software to predict the (B) cellular localization of fast and slow synthesis secreted proteins of PHT cells.

**Supplemental Figure 3**. **Gene Ontology (GO) analysis of fast and slow synthesis proteins of Primary Human Trophoblast secretome**. (A) The identified fast and slow synthesis proteins in the PHT secretome were analyzed by Functional Enrichment Analysis Tool ([www.funrich.org](http://www.funrich.org)), a subcellular localization predication software to predict the (B) biological process of fast and slow synthesis secreted proteins of PHT cells.

**Supplemental Figure 4**. **Granulin and fibronectin proteins in PHT conditioned media validated by Western blotting.** Validation of results from SILAC‐based secretome by Western blotting. Representative western blots of granulin and fibronectin expression in PHT cells conditioned media. Equal loading was performed. Identification of these proteins in PHT cell conditioned media using immunoblotting was consistent with the results from our SILAC approach. The lower panel shows the Ponceau stain of the blot to confirm equal protein loading.

**Supplemental Figure 5. Vesicular integral-membrane protein (Vip36) and Azurocidin protein expression in placental homogenates.** Validation of results from SILAC‐based secretome by Western blotting. To add confidence in our SILAC secretome data, placental expression of the (A) vesicular integral-membrane protein (Vip36) and (B) azurocidin proteins by immunoblotting using placental homogenates. Representative western blots of vesicular integral-membrane protein (Vip36) and azurocidin expression in placental homogenates. Equal loading was performed. Identification of these proteins in placental homogenates using immunoblotting was consistent with the results from our SILAC approach. The lower panel shows the Ponceau stain of the blot to confirm equal protein loading.

**Supplemental Figure 6**. **Culturing PHT cells in SILAC media does not affect trophoblast cleaved caspase-3 expression (apoptosis).** (A) Cultured primary trophoblast cells were incubated in DMEM + F12 media with stable isotope-labeled lysine and arginine (Lys4/Arg6) or DMEM + F12 media with unlabeled lysine and arginine. Representative western blot of cleaved caspase-3 cell lysates of DMEM + F12 media with stable isotope-labeled lysine and arginine (Lys4/Arg6) or DMEM + F12 media with unlabeled lysine and arginine cells. Equal loading was performed. The lower panel shows the Ponceau stain of the blot to confirm equal protein loading. (B) Histogram summarizes the relative density expression of cleaved caspase-3 in DMEM + F12 media with stable isotope-labeled lysine and arginine (Lys4/Arg6) or DMEM + F12 media with unlabeled lysine and arginine. Values are mean + SEM for cultured PHT cells isolated from four different placentas.
