# Supplementary material for: Characterization of the Primary Human Trophoblast Cell Secretome Using Stable Isotope Labeling With Amino Acids in Cell Culture
Source: Front Cell Dev Biol. 2021 Sep 14;9:704781. doi: 10.3389/fcell.2021.704781 (PMC8476785; doi:10.3389/fcell.2021.704781)
Supplement: Supplementary file 3 [file Data_Sheet_2.docx]

**Supplemental Methods**

**Isolation and culture of primary human trophoblast cells**

Healthy women with normal term pregnancies (>37 weeks of gestation) delivered by Caesarean section were recruited following written informed consent. Selected clinical characteristics of the study subjects are provided in supplemental Table 5. In brief, cells were cultured in DMEM: F12 (1:1) media for SILAC (Thermo Fisher Scientific) containing 10 % of dialyzed fetal bovine serum (Thermo Fisher Scientific), 50 μg/ml gentamicin, 60 μg/ml benzylpenicillin and 100 μg/ml streptomycin. PHT cells were plated at a density of 2.75 million in 35-mm dishes and incubated in a 5 % CO_2_ humidified atmosphere at 37 °C. Over a few days in culture, active mononucleated cytotrophoblast cells fuse into multinucleated syncytiotrophoblast cells (1), and begin secreting chorionic gonadotropin (hCG) (2). We and others previously demonstrated that nearly all cytotrophoblast cells differentiate into multinucleated syncytiotrophoblast over 72 h in culture, as evidenced by markedly increased hCG secretion (3-5). In addition, we have also shown that the expression of syncytin protein (a fusion protein that plays a significant role in cytotrophoblast fusion) increases over the culture period, further confirming differentiation of isolated villous cytotrophoblast cells into syncytiotrophoblasts (6). The cells were cultured for a total of 114 h with change of the culture media every 24 hr. To confirm cell differentiation, release of human chorionic gonadotropin (hCG) into the cell culture media was assessed with a commercial ELISA kit (Immuno-Biological Laboratories, Minneapolis, MN) at 18, 66, 90, 114 h after plating the cells.

**Collection of conditioned media and processing**

At 114 h and following 24 h culture in serum-free media, the conditioned media from PHT cells was collected and centrifuged at 800 × g for 10 min to remove any detached cells floating in the medium. The supernatant was filtered using a 0.22-µm filter. The filtered supernatant was subsequently concentrated using 3-kDa cut-off filters (Millipore, Billerica, MA, USA). Protein concentration was measured using bicinchoninic acid (BCA, Bio Rad, CA).

**Western blotting**

Western blotting was carried out as described (7). Protein expression of syncytin and caspase-3 was analyzed in cell lysates to assess syncytialization and presence of apoptosis, respectively. In brief, 5-10 μg of total protein was loaded onto a NuPAGE Novex (Invitrogen, Carlsbad, CA, USA) precast 4-12% Bis-Tris gel and electrophoresis was performed at a constant 200 V for 40 min. Proteins were transferred onto nitrocellulose membranes at a constant 40 V. After transfer, membranes were blocked in 5% milk in Tris-buffered saline (w/v) plus 0.1% Tween 20 (v/v) for 1 h at room temperature. Membranes were incubated with primary antibodies overnight at 4 °C. Subsequently, membranes were incubated with the appropriate peroxidase-labeled secondary antibodies for 1 h. After washing, bands were visualized using enhanced chemiluminescence detection reagents (Pierce Biotechnology, Rockford, IL, USA). Blots were stripped using β-mercaptoethanol and re-probed for β-actin as a loading control. For the conditioned media, blots were probed with Ponceau stain to confirm equal protein loading.

**Protein analysis by mass spectrometry**

Proteins retained by the 3-kDa spin columns were separated by 1-D SDS-PAGE using a Criterion XT 12% gel that was electrophoresed for 2 cm and then stained with Coomassie blue. The protein-containing region of each gel lane was divided into six slices~~,~~ which were individually reduced *in situ* with TCEP [tris(2-carboxyethyl) phosphine] and alkylated in the dark with iodoacetamide prior to treatment with trypsin (sequencing grade; Promega). The digests were analyzed by capillary HPLC-electrospray ionization tandem mass spectrometry (HPLC-ESI-MS/MS) on a Thermo Fisher LTQ Orbitrap Velos Pro mass spectrometer fitted with a New Objective Digital PicoView 550 NanoESI source. On-line HPLC separation of the digests was accomplished with an Eksigent/AB Sciex NanoLC-Ultra 2-D HPLC system: column, PicoFrit™ (New Objective; 75 μm i.d.) packed to 10 cm with C18 adsorbent (Vydac; 218MSB5, 5 μm, 300 Å); mobile phase A, 0.5% acetic acid (HAc)/0.005% trifluoroacetic acid (TFA); mobile phase B, 90% acetonitrile/0.5% HAc/0.005% TFA; gradient 2 to 42% B in 30 min; flow rate, 0.4 μl/min. Precursor ions were acquired in the Orbitrap from m/z 300 – 2,000 in profile mode at 60,000 resolution (*m/z* 400); data-dependent collision-induced dissociation spectra of the six most intense ions in the precursor scan above an intensity threshold of 3,000 were acquired at the same time in the linear trap (30% normalized collision energy). Singly charged and ions with unassigned charge states were rejected. Dynamic exclusion settings were repeat count, 1; repeat duration, 30 sec; exclusion duration, 30 sec; exclusion limit, 500 entries.

**MS Data Processing**

After peak list generation by Mascot Distiller (Matrix Science) for each MS data file, Mascot (Matrix Science) was used to search the human subset of the UniProt database [Uniprot_Human 20181204 (95,936 sequences: 38,067,061 residues)] and a database of common contaminants (20120713, 247 sequences, 128,130 residues). Search parameters for protein identification were: precursor mass tolerance, ± 20 ppm (# ^13^C = 1); product ion mass tolerance, ± 0.8 Da; fixed modification, carbamidomethylation (C); variable modifications, deamination (NQ), oxidation (M), ^2^H_4_ (K), ^13^C_6_ (R); enzyme, trypsin, with one missed cleavage allowed. Subset search of the identified proteins by X! Tandem, cross-correlation with the Mascot results and determination of protein and peptide identity probabilities were accomplished by Scaffold 4 (Proteome Software). The thresholds for acceptance of peptide and protein assignments in Scaffold were set to 99% protein, 1 minimum peptide, 99.9% peptide to achieve a protein level FDR of < 1%. For assessment of incorporation of stable isotope labels, the searches were configured in Mascot for two component SILAC (SILAC K+4_R+6 [MD]); carbamidomethylation (C) was set as a fixed modification as and no ^13^C was included in the precursor mass tolerance setting. The MS and Mascot data files for each gel lane were then imported into Mascot Distiller and processed as a multi-file project for determination of relative protein quantities based on the intensities of the labeled and unlabeled peptide precursor ions. The following conservative Distiller quantitation thresholds were used: correlation, 0.9; standard error, 0.15; fraction, 0.5; a peptide had to be assigned above the homology threshold to be considered for quantitation. A minimum of two peptides was required.

PHT secretome was subjected to gene ontology (GO) functional annotation analysis under biological processes and molecular function category using DAVID Bioinformatics Resources to determine significantly enriched genes. ExoCarta database was used to determine exosomal proteins.

**Measurement of Serum Legumain**

We quantified the abundance of legumain in paired serum samples collected from women during her pregnancy (36 weeks of pregnancy) and postpartum (3rd week of postpartum). Legumain in the serum was measured with a commercial ELISA kit (Invitrogen Thermo Scientific, Carlsbad, CA; Product # EH299RB).

**Culturing PHT cells in SILAC media does not affect trophoblast cell viability, differentiation, or apoptosis.**

Following isolation and plating, trophoblast cells aggregate and fuse to become multinucleated, a process resembling syncytiotrophoblast formation *in vivo*. Cultured PHT cells can be used, therefore, as a model to study the syncytiotrophoblast, the transporting and hormone-producing epithelium of the human placenta. After 66 h in culture, there was a marked increase in hCG production by trophoblast cells, and the levels remained high until at least 114 h after plating (Supplemental Figure 1A). Because hCG is produced predominantly by the syncytiotrophoblast, these data provide evidence of cell differentiation and syncytialization. Furthermore, hCG secretion profiles in cells cultured in SILAC media with labeled K4R6 were similar to cells cultured in DMEM + F12 media with unlabeled lysine and arginine (Supplemental Figure 1A). We also found that there was no significant difference in the protein expression of the apoptosis marker caspase-3 (Supplemental Figure 1B) or in differentiation marker syncytin (Supplemental Figure 1C) in trophoblast cells cultured in SILAC media with labeled lysine and arginine (K4R6) as compared to cells cultured in DMEM + F12 media with unlabeled amino acids.

In addition, we have measured the LDH (LDH kit, Cambridge, United Kingdom), a reliable marker of cellular toxicity. It has been demonstrated that higher levels of LDH are rapidly released into the cell culture conditioned media when the plasma membrane of the cell is damaged (8). LDH leakage in conditioned media of PHT cells cultured in serum-free media (LDH: 1.1± 0.3 mIU/ml, n=4) and complete media (with serum, LDH: 1.3 ± 0.3 mIU/ml, n=4) were comparable. Our results indicate that culturing PHT cells in serum-free media for a period of 24 h did not induce cellular toxicity and increase membrane permeability and apoptotic cell degradation.

**Validation of results from SILAC‐based secretome by Western blotting**

We further validated secreted protein (granulin, fibronectin) in PHT cell-conditioned media by Western blotting as described above (Supplemental Figure 4). Identification of these proteins in PHT cell-conditioned media using immunoblotting was consistent with the results from our SILAC approach. Furthermore, we confirmed the placental expression of vesicular integral-membrane protein (Vip36) and azurocidin proteins by immunoblotting using placental homogenates (Supplemental Figure 5 ) to add confidence in our SILAC secretome data. We have previously reported protein expression of vinculin, eIF2 alpha, PI3 kinase p85, p44/42 MAPK ERK1/2, STAT3 in the human placenta (9, 10). All these proteins are secreted by PHT cells. (Supplemental Table1).

**Culturing PHT cells in SILAC media does not affect trophoblast cleaved caspase-3 expression**

In addition, we demonstrated that there was no significant difference in the protein expression of the cleaved caspase-3 (Supplemental Figure 6) in trophoblast cells cultured in SILAC media with labeled lysine and arginine (K4R6) as compared to cells cultured in DMEM + F12 media with unlabeled amino acids. Apoptosis stimulus is known to increase the cleaved caspase-3 expression in trophoblast cells (11). This data suggest that culturing of trophoblast cells in SILAC media with labeled lysine and arginine (K4R6) did not induce apoptosis.

**References**

1. Kliman, H. J.; Segel, L., The placenta may predict the baby. *J Theor Biol* **2003,** 225, (1), 143-5.

2. Bloxam, D. L.; Bax, B. E.; Bax, C. M., Culture of syncytiotrophoblast for the study of human placental transfer. Part II: Production, culture and use of syncytiotrophoblast. *Placenta* **1997,** 18, (2-3), 99-108.

3. Li, L.; Schust, D. J., Isolation, purification and in vitro differentiation of cytotrophoblast cells from human term placenta. *Reprod Biol Endocrinol* **2015,** 13, 71.

4. Rosario, F. J.; Kanai, Y.; Powell, T. L.; Jansson, T., Mammalian target of rapamycin signalling modulates amino acid uptake by regulating transporter cell surface abundance in primary human trophoblast cells. *J Physiol* **2013,** 591, 609-625.

5. Jansson, T.; Castillo-Castrejon, M.; Gupta, M. B.; Powell, T. L.; Rosario, F. J., Down-regulation of placental Cdc42 and Rac1 links mTORC2 inhibition to decreased trophoblast amino acid transport in human intrauterine growth restriction. *Clin Sci (Lond)* **2020,** 134, (1), 53-70.

6. Chen, Y. Y.; Powell, T. L.; Jansson, T., 1,25-Dihydroxy vitamin D3 stimulates system A amino acid transport in primary human trophoblast cells. *Mol Cell Endocrinol* **2017,** 442, 90-97.

7. Kavitha, J. V.; Rosario, F. J.; Nijland, M. J.; McDonald, T. J.; Wu, G.; Kanai, Y.; Powell, T. L.; Nathanielsz, P. W.; Jansson, T., Down-regulation of placental mTOR, insulin/IGF-I signaling, and nutrient transporters in response to maternal nutrient restriction in the baboon. *FASEB J* **2014,** 28, (3), 1294-305.

8. Uchide, N.; Ohyama, K.; Bessho, T.; Toyoda, H., Lactate dehydrogenase leakage as a marker for apoptotic cell degradation induced by influenza virus infection in human fetal membrane cells. *Intervirology* **2009,** 52, (3), 164-73.

9. Keleher, M. R.; Erickson, K.; Smith, H. A.; Kechris, K. J.; Yang, I. V.; Dabelea, D.; Friedman, J. E.; Boyle, K. E.; Jansson, T., Placental Insulin/IGF-1 Signaling, PGC-1alpha, and Inflammatory Pathways Are Associated With Metabolic Outcomes at 4-6 Years of Age: The ECHO Healthy Start Cohort. *Diabetes* **2021,** 70, (3), 745-751.

10. Keleher, M. R.; Erickson, K.; Kechris, K.; Yang, I. V.; Dabelea, D.; Friedman, J. E.; Boyle, K. E.; Jansson, T., Associations between the activity of placental nutrient-sensing pathways and neonatal and postnatal metabolic health: the ECHO Healthy Start cohort. *Int J Obes (Lond)* **2020,** 44, (11), 2203-2212.

11. Kim, M. J.; Kim, C. H.; An, M. J.; Lee, J. H.; Shin, G. S.; Song, M.; Kim, J. W., Ethylparaben induces apoptotic cell death in human placenta BeWo cells via the Caspase-3 pathway. *Anim Cells Syst (Seoul)* **2020,** 24, (1), 34-43.
