# Supplementary figures and images for: Characterization of the Primary Human Trophoblast Cell Secretome Using Stable Isotope Labeling With Amino Acids in Cell Culture
Source: Front Cell Dev Biol. 2021 Sep 14;9:704781. doi: 10.3389/fcell.2021.704781 (PMC8476785; doi:10.3389/fcell.2021.704781)

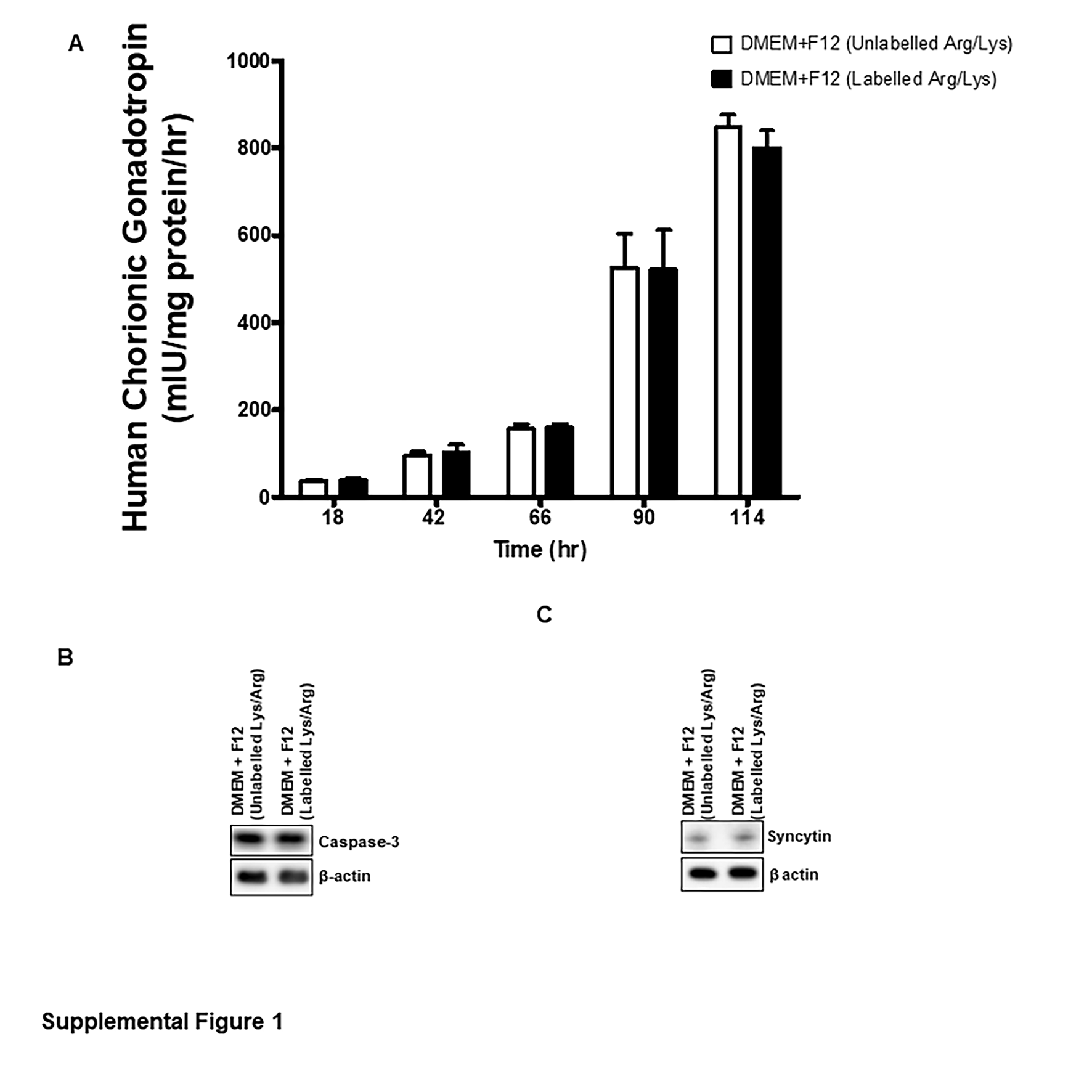

Supplement: Supplementary file 4 [file Image_1.tif]

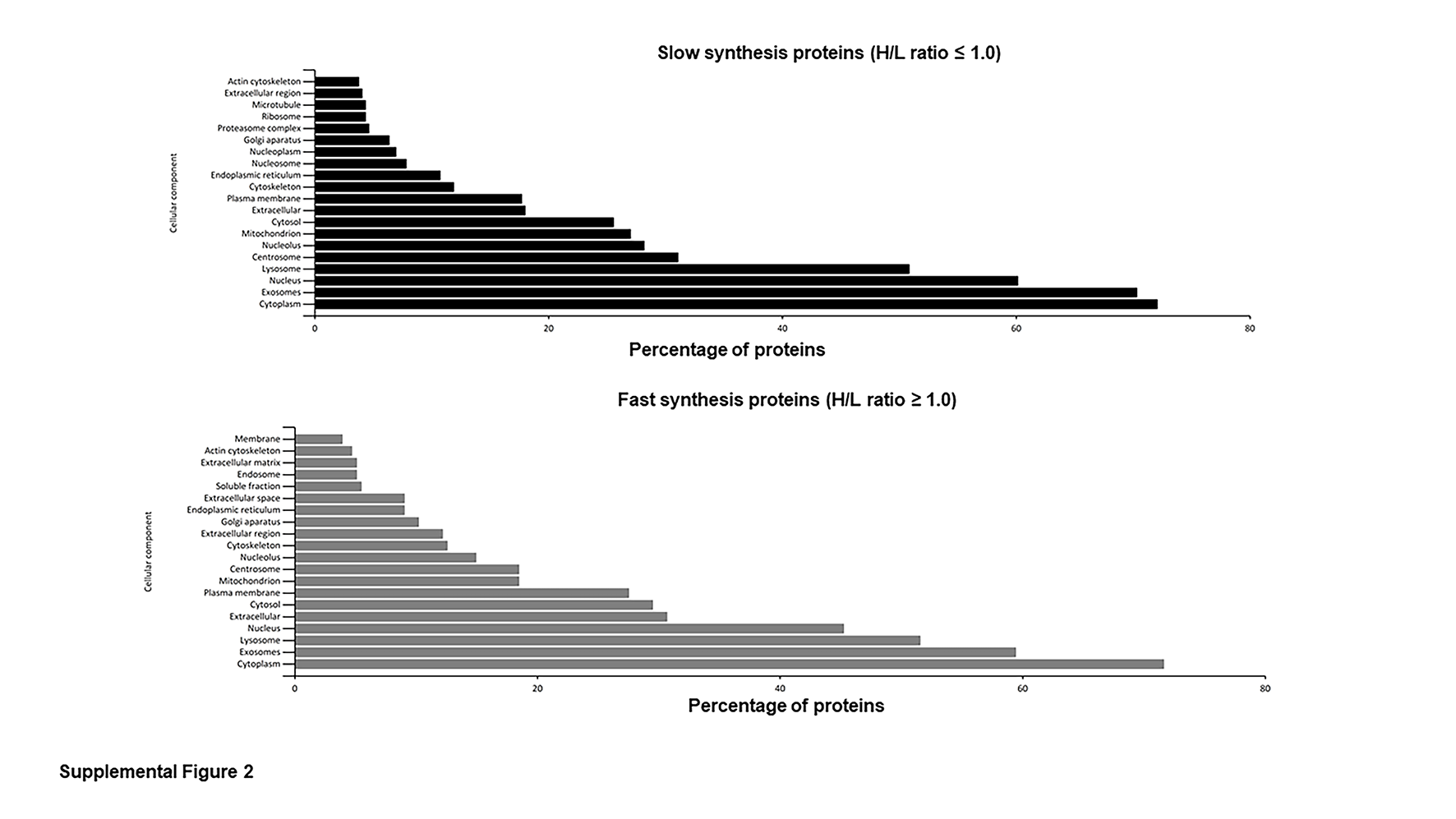

Supplement: Supplementary file 5 [file Image_2.tif]

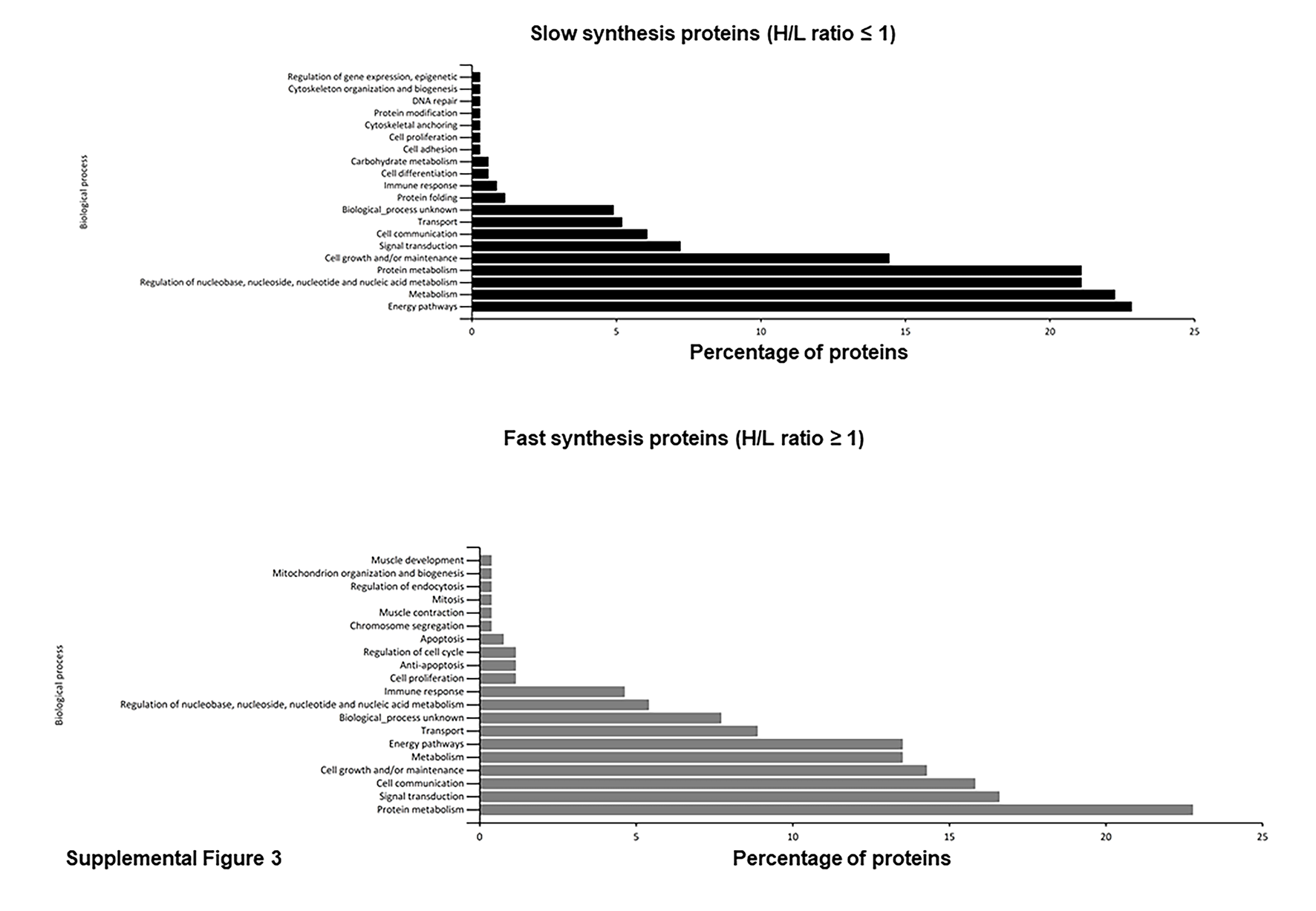

Supplement: Supplementary file 6 [file Image_3.tif]

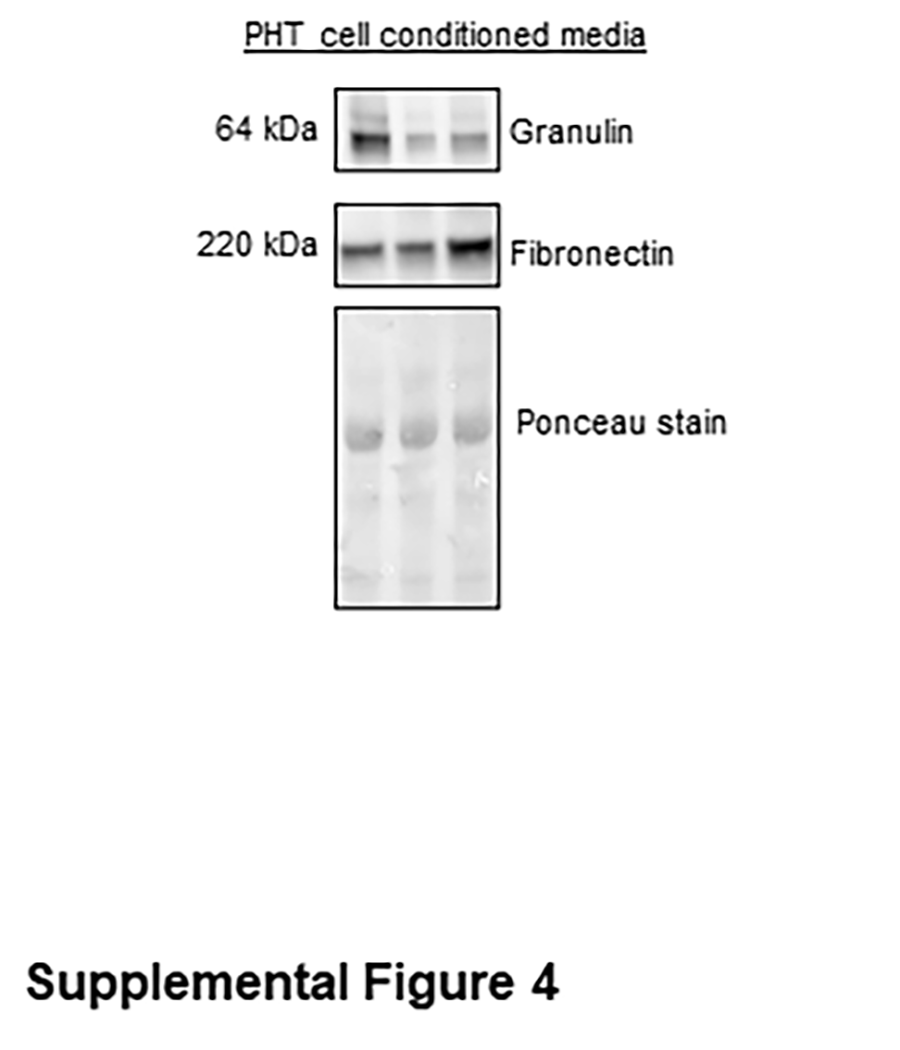

Supplement: Supplementary file 7 [file Image_4.tif]

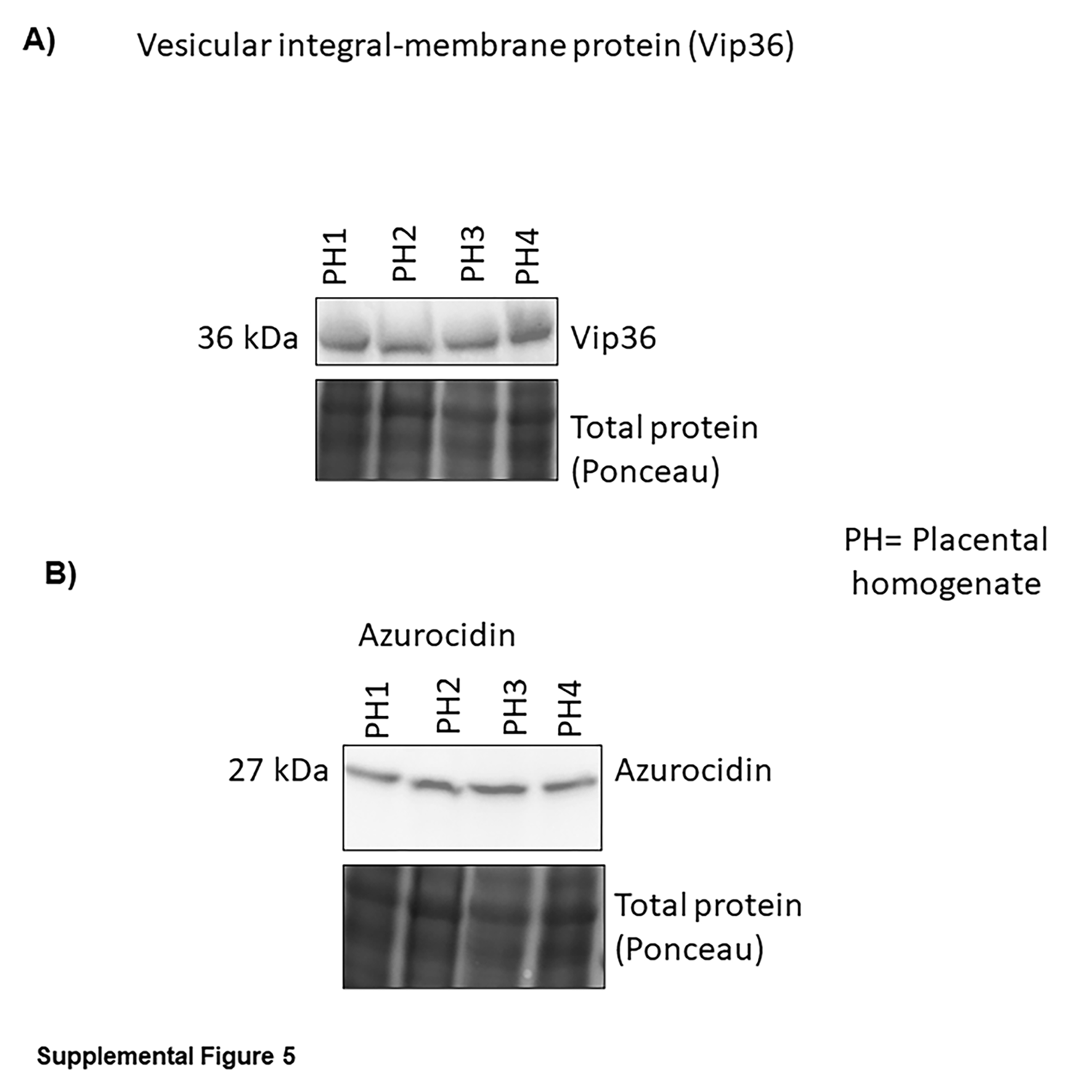

Supplement: Supplementary file 8 [file Image_5.tif]

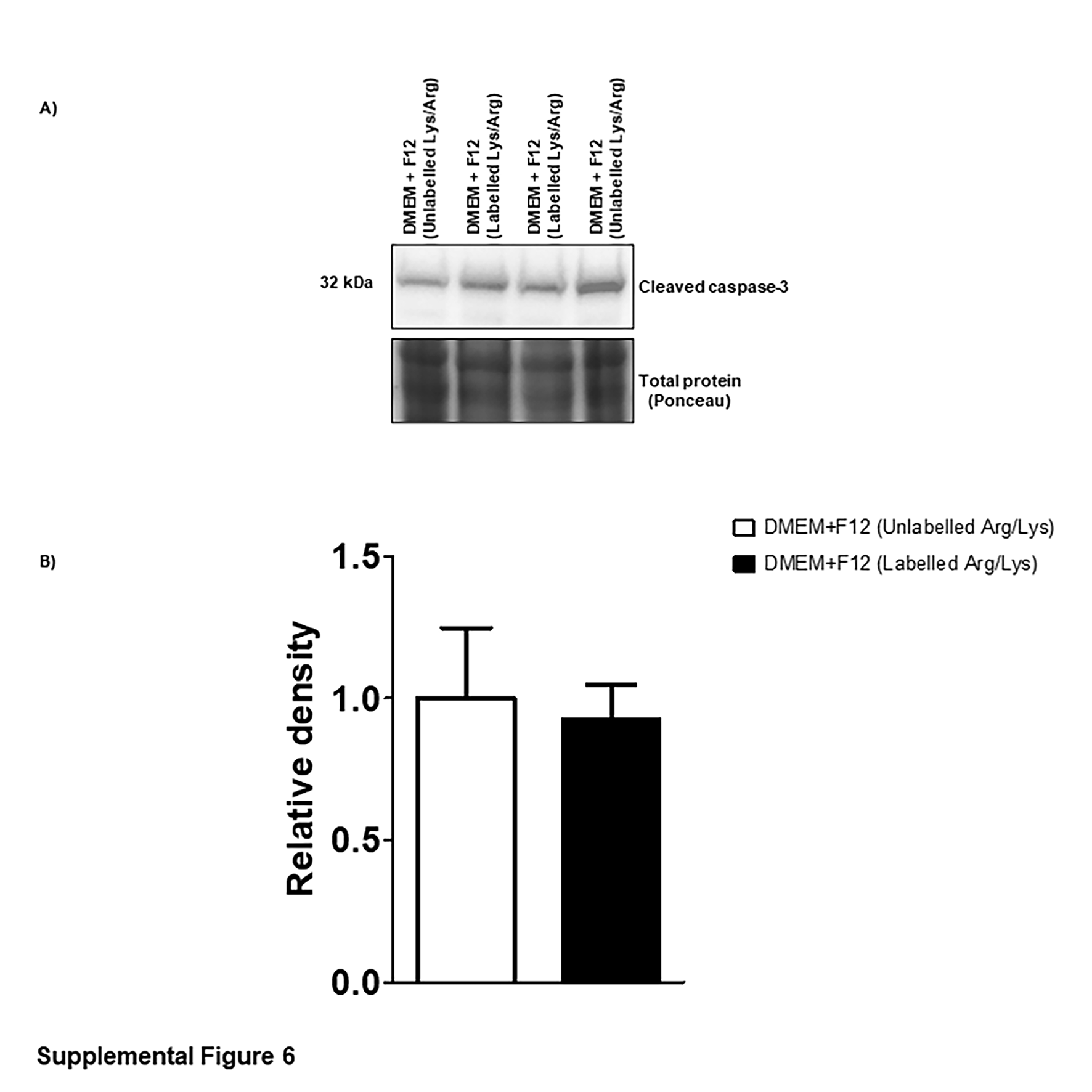

Supplement: Supplementary file 9 [file Image_6.tif]
